# Topiramate-induced bilateral central serous chorioretinopathy

**DOI:** 10.5935/0004-2749.2022-0243

**Published:** 2025-02-11

**Authors:** Alparslan Şahin, Fatih Mehmet Türkcü, Yasin Çınar

**Affiliations:** 1 Private Bower Hospital, Department of Ophthalmology, Diyarbakir, Turkey; 2 Department of Ophthalmology, School of Medicine, Dicle University, Diyarbakir, Turkey

To the Editor,

Central serous chorioretinopathy (CSCR) is characterized by fluid acumination in the subretinal space. Several risk factors such as pregnancy, type A personality, smoking, psychological stress, and medications are associated with CSCR^(1)^. Topiramate is an antiepileptic drug that is also used for migraine prophylaxis. Its ocular side effects includes myopic shift, acute angleclosure glaucoma, and choroidal effusion^(2)^. Anterior segment complications of topiramate are more frequent than posterior ocular involvement. However, cases with CSCR-like findings have been reported recently^(3,4)^. Herein, we present a case of CSCR that developed after topiramate treatment that resolved following drug cessation.

**Figure 1:**
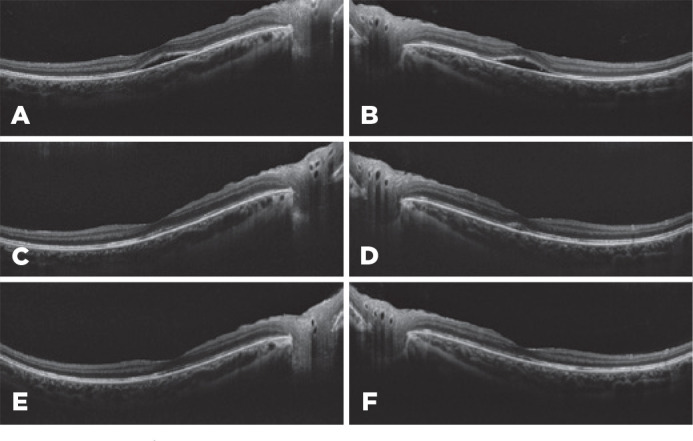
Optical coherence tomography images of the patient on admission (A, B) and first (C, D) and second (E, F) months after admission. The serous retinal elevation completely recovered 2 months after admission. CSCR that resolved after medication cessation. Although anterior segment side effects have been frequently reported, topiramate may also affect the posterior segment of the eye. Rosenberg et al. reported two cases of macular neurosensory retinal detachment secondary to topiramate use^(4)^. One patient had bilateral, and the other had unilateral serous detachment. However, their case with bilateral involvement was on topical steroids during the symptoms. In addition, Mazumdar et al. reported a case of unilateral serous retinal detachment^(3)^. Additionally, their case was under topical steroid treatment. These case reports showed that cessation of topiramate leads to the recovery of serous detachment.

A 22-year-old female patient was admitted to the ophthalmology clinic with a complaint of loss of central vision. Her visual acuity was 7/10 bilateral in the Snellen chart. Her intraocular pressure was 15/15 mmHg. Anterior segment examination was unremarkable. Dilated fundus examination revealed bilateral apparent serous elevation of the central retina and pigment epithelial alterations. Optical coherence tomography (OCT) images revealed bilateral central serous detachment of the macula (Figures 1A-B). She had a history of headache, and she was on a 50 mg topiramate (Topamax; Ortho-McNeil Neurologics, Titusville, NJ) treatment twice a day for her complaint for 3 weeks. We considered possible serous detachment of the macula secondary to topiramate treat ment, and we discontinued topiramate medication. We did not perform fluorescein angiography because the patient declined it. Additionally, topical treatment of brinzolamide 1% (Azopt) twice a day and nepafenac 0.1% (Nevanac, Alcon Labs, Fort Worth, TX, USA) four times a day were given.

One month later, her visual acuity increased to 8/10 bilaterally. Fundus examination showed retinal pigment epithelial alterations. OCT demonstrated minimal subretinal fluid (Figure 1C-D). She continued the topical eye drops. Two months after the first examination, her visual acuity improved to 10/10 bilaterally. Fundus examination was unremarkable, and OCT images revealed normal anatomy of the retina (Figure 1E-F).

Several medications such as steroids, phosphodiesterase inhibitors, etc., are risk factors for CSCR development^(1)^. This case revealed that topiramate may cause

Clinicians should consider the possible effects of topiramate treatment on the pathogenesis of CSCR. Topiramate cessation may result in the regression of the serous detachment of the retina. Since a prompt diagnosis is important to prevent further damage to the retina, both ophthalmologists and neurologists should be aware of serous retinal detachment after topiramate treatment.
